# Transcriptional networks weaving natural killer anti-tumor immune response

**DOI:** 10.1186/s12943-026-02643-4

**Published:** 2026-03-11

**Authors:** Anna Rita Redavid, Martina Bigliardi, Alessia Ciarrocchi, Francesca Reggiani

**Affiliations:** Translational Research Laboratory, Azienda USL-IRCCS di Reggio Emilia, Viale Risorgimento 80, Reggio Emilia, 42123 Italy

**Keywords:** Natural killer, Transcription factor, Tumor ecosystem, Immunotherapy

## Abstract

**Background:**

Natural killer cells (NKs) are critical effectors of innate immune surveillance, capable of eliminating target cells without prior antigen sensitization. The crucial role of NKs in cancer immunity has largely been highlighted in both hematological and solid tumors. Besides, NK-based cell therapies have gained momentum as a compelling alternative to T cell-based approaches, due to their off-the-shelf availability and lower risk of toxicity. Still, the intrinsic molecular mechanisms driving NK anti-tumor efficacy in different tumor contexts are poorly described, drastically impairing their clinical exploitation.

**Aims:**

This review aims to provide a comprehensive overview of the transcriptional networks defining each step of NK anti-tumor response, from tissue recruitment to target recognition and cytotoxic activation. The molecular mechanisms triggering NK dysfunction in the tumor microenvironment are also highlighted from a transcriptional perspective. The described time- and context-dependent transcriptional machinery is characterized by a constant interplay between activators and repressors, which integrates and balances signals deriving from the surrounding tumor ecosystem.

**Conclusions:**

Considering the relevance of transcription factors in controlling NK functions, their potential exploitation as novel therapeutic targets, through either pharmacological approaches or genome editing, is a new opportunity for cancer treatment.

## Introduction

Natural killer cells (NKs) are essential players of tumor immunosurveillance, capable of eliminating transformed cells independently of antigen presentation or major histocompatibility complex class-I (MHC-I) restriction. Their primary effector functions include the release of lytic granules, containing cytotoxic molecules such as perforins and granzymes, and the secretion of cytokines that modulate the surrounding tumor immune ecosystem. However, insufficient tissue infiltration and the immunosuppressive nature of the tumor microenvironment (TME) severely impair NK functions. These limitations reduced tumor recognition and clearance, restraining NK clinical application.

To augment the therapeutic potential of NKs, a variety of drugs, cytokines, and antibodies is being developed to enhance their effector functions [1]. Furthermore, the engineering of NK surface receptors has improved tumor recognition and NK delivery in adoptive cell transfer. Chimeric antigen receptor (CAR)-NK-based cell therapies have expanded clinical applications, offering a promising alternative with an superior safety profile compared to CAR-T cells [2]. Nevertheless, the partial knowledge of intracellular and extracellular mechanisms governing NK activation restrains the proper stimulation of their anti-tumor activity across most clinical settings.

An emerging area of investigation focuses on the transcriptional landscapes that orchestrate NK cell activity in different tumor contexts. Recent studies employing single-cell RNA sequencing (scRNA-seq) have extensively profiled NK subsets in distinct compartments, including peripheral blood, tissues, and tumors, revealing that each is characterized by distinct gene regulatory networks and dominant transcription factors (TFs) [3–5]. While the transcriptional axes governing NK differentiation under homeostatic conditions have been well elucidated [6], recent evidence demonstrated that the effector state of NKs is intricately and dynamically modulated by distinct TFs [3]. Tumor-infiltrating NKs (TiNKs) have been stratified based on specific gene expression profiles and TFs, which define their transition from the less cytotoxic CD56^bright^ phenotype toward the more mature and cytotoxic CD56^dim^ state.

This review aims to elucidate the transcriptional networks and associated TFs regulating NK functions, with a specific focus on the tumor ecosystem. We highlight the recent findings and advancements in the molecular mechanisms governing NK recruitment, activation, and anti-tumor responses. Furthermore, the dysfunctional processes leading to intrinsic NK cell exhaustion during tumor progression are described from a transcriptional perspective. Finally, we provide a comprehensive overview of how modulating TF activity represents a novel therapeutic frontier to enhance NK-based immunotherapies in cancer treatment.

### Transcriptional regulation of NK trafficking and tumor infiltration

NK trafficking and homing in different tissues are precisely orchestrated processes, governed by the interplay of surface receptors and adhesion molecules. Specifically, the expression of integrins allowed for the distinction between functionally circulating conventional NK (cNK) and tissue-resident NK (trNK) populations [7–9]. The differential expression of CXC-motif chemokine receptors (CXCR) modulates the responsiveness of NKs to specific C-C motif chemokine ligand (CCL) and C-X-C motif chemokine ligand (CXCL), thereby influencing their trafficking potential and niche retention.

NK trafficking and tumor recruitment are governed by distinct transcriptional networks that regulate the surface expression of key molecules, including integrins, selectins, chemokine receptors, and tissue-egress factors. These regulatory axes influence migratory surveillance and niche retention, both of which are pivotal for an effective NK-mediated anti-tumor response.

#### NK cell heterogeneity and residency features

NKs exhibit significant heterogeneity regarding their functional properties and developmental ontogeny. Traditionally, their classification has relied on the differential expression of surface markers. Human NKs are primarily divided into two distinct subsets based on the surface density of CD56: the CD56^bright^ population, typically characterized as less mature and cytotoxic, and the CD56^dim^ subset, which represents terminally differentiated cells with higher cytolytic potential.

The emergence of single-cell technologies and advanced computational pipelines has challenged the traditional paradigm, extending the classification beyond the CD56-metrics [4]. Within peripheral blood, cNKs were categorized into three main clusters (NK1, NK2, and NK3), each defined by distinct transcriptional features.

The NK1 cluster was characterized by robust expression of CD16, CX3CR1, CD161, and CD38, but low levels of CD56. The NK2 subgroup exhibited sustained expression of CD56, CD27, CD44, NKp46, and NKG2D, while lacking CD16. The NK3 cluster was characterized by the presence of CD16, CD57, and CD49a, coupled with low CD56.

These three major populations were further divided into 6 subgroups (NK1A, NK1B, NK1C, NKint, NK2, NK3). TFs governing NK differentiation, such as T-BET and B-lymphocyte-induced maturation protein-1 (BLIMP1, *PRDM1*), exhibited a progressive increase from the NK2 toward the NK1 subset. Conversely, NK2 cells displayed higher expression of MYC, TCF1, RUNX2, and GATA-binding protein 3 (GATA3), whereas NK3 cells maintained sustained level of ASCL2, KLF6, and BLIMP1.

The classical distinction of cNKs and trNKs is based on the differential expression of integrins, selectins, and chemokine receptors that confer them specific residency features [8, 10]. Human trNKs are characterized by the expression of CD69, CD49a, CD103, and CXCR6, which facilitate their tissue retention. Conversely, cNKs express a distinct repertoire of markers, including CX3CR1, C-C Motif Chemokine Receptor 7 (CCR7), CD62L, and CD49e, enabling their systemic recirculation.

Single-cell analysis across healthy tissues and 22 distinct tumor types has recently elucidated the distribution pattern of trNK subsets [4]. In homeostatic tissues, trNK signatures consistently aligned with the NK1 and NK2 blood subgroups, corresponding to the CD56^dim^ and CD56^bright^ populations, respectively. However, a relevant transcriptional shift occurred in the TME, indicating a specific tumor effect in modulating the NK transcriptome. Notably, a systemic enrichment of NK2 cells was observed within the TME compared to healthy tissues.

This phenotypic heterogeneity was further corroborated by previous investigations, which identified tissue-residency signatures and niche-specific adaptations within immature CD56^bright^ NKs, while failing to detect comparable transcriptional variability in circulating CD56^dim^ mature populations [11]. In human lungs, CD56^bright^ NKs constituted a heterogeneous population exhibiting hallmark features of tissue residency, including CD49a and CD103, while being functionally competent [12]. These lung trNKs were characterized by elevated transcriptional activity of HOBIT and reduced levels of the Krüppel-like factors (KLF) KLF2 and KLF3. Comparative gene expression profiles of trNKs isolated from the lungs and bone marrow revealed that the local microenvironments imprinted these cells with distinctive features.

Tissue-specific transcriptional landscapes were further elucidated in murine models [13]. A CD49a^+^ cluster associated with the lamina propria was defined by high levels of the TF HELIOS and the orphan nuclear factor NR4A1. In salivary glands, two clusters characterized by sustained CD49a and intermediate CD49b expression displayed a similar phenotype to innate lymphoid cells (ILCs). Conversely, splenic and hepatic specific clusters, marked by high CD49b and CD122 expression without CD49a, exhibited enriched levels of cytotoxic genes and specific TFs, KLF2 and IRF8, being formerly associated with circulating NKs.

Collectively, these findings suggest that different NK subsets coexist within each tissue, including both tissue-non-specific circulating populations and specific trNKs with distinctive transcriptional features.

It has been proposed that, in certain tissues, trNKs do not derive from circulating cNKs but instead differentiate from a distinct lineage of in situ progenitors [14, 15]. In mice, the immature phenotype of hepatic trNKs (CD49a^+^ DX5^−^ CD27^+^ CD11b^low^ TRAIL^+^) was distinct from both thymic CD127^+^ NKs, which relied on GATA3 expression, and splenic CD49a^−^ DX5^+^ cNKs, which required E4BP4 (*Nfil3*) [14]. Functionally, trNKs contributed to local inflammation by secreting abundant tumor necrosis factor (TNF)α and granulocyte-macrophage colony-stimulating factor (GM-CSF), whereas cNKs primarily released interferon (IFN)γ. Transcriptomic profiling has further confirmed that hepatic trNKs did not represent a transitioning immature stage toward cNKs, but rather constituted a separate lineage characterized by reduced EOMES levels despite comparable T-BET expression [14]. Notably, while T-BET expression was required for the hepatic trNK development, their ontogeny remained independent of E4BP4, which specifically governed cNK maturation. Similar trNK phenotypes have been identified in other solid organs, such as the uterus and skin [14]. Furthermore, the extramedullary origin of NKs from in situ progenitors has been reported in secondary lymphoid tissues, including tonsils and lymph nodes [15].

#### NK trafficking

The homing properties of NKs are intrinsically associated with their differentiation state, which is orchestrated by a network of pivotal developmental TFs. Within this regulatory framework, the T-box family members, EOMES and T-BET (*Tbx21*), emerge as central mediators of both NK differentiation and trafficking potential. EOMES is enriched in immature NKs, whereas T-BET upregulation is a hallmark of NK terminal differentiation [16]. Chromatin immunoprecipitation sequencing (ChIP-seq) data have revealed a substantial overlap in the binding profile of these TFs. In murine models, the frequency of cNKs was markedly reduced upon the combined deletion of T-BET and EOMES, primarily due to the downregulation of the egress factor sphingosine-1-phosphate receptor (S1PR)-5, a common shared transcriptional target. Conversely, the modulation of each TF yielded distinct gene expression profiles, suggesting that maturation-specific epigenetic marks or cofactors governed their chromatin accessibility. In T-BET-deficient mice, NKs were depleted in the peripheral blood but remained stable in the bone marrow [16], confirming the requirement of T-BET for promoting NK circulation [17]. Nevertheless, certain trafficking-related genes were identified as EOMES-specific targets, including the integrin CD11B (*Itgam*) and the homing receptor CD62L (*Sell*) [16], further supporting a complementary role of EOMES in the regulation of NK tissue residency.

Beyond the T-box family, members of the Forkhead box O (FOXO) protein family serve as critical drivers of NK differentiation and homing. NKs predominantly express FOXO1 and, to a lesser extent, FOXO3, while lacking FOXO4 and FOXO6 [18]. In mice carrying the genetic deletion of *Foxo1* or *Foxo3*, NKs were selectively depleted within lymph nodes. Consistent with these observations, the loss of FOXO1 and FOXO3 reduced CD62L expression, thereby restraining NK trafficking potential. This regulatory axis may be further modulated by the aryl hydrocarbon receptor (AHR), which, upon ligand engagement, impaired CD62L expression by stimulating ADAM17 metallopeptidase activity [19]. Consequently, the absence of AHR abrogated the capacity of NKs to migrate and infiltrate tumors [20].

Furthermore, FOXO1 has been reported to bind the T-BET (*Tbx21*) promoter, repressing its transcription and providing a mechanistic explanation for the observed impairment in cNK trafficking [18].

In addition to T-box and FOXO factors, NK tissue residency was regulated by **RUNX2**, a member of the highly conserved runt-related transcription factor (RUNX) family [21]. Knockdown (KD) of RUNX2 decreased tissue residency-specific markers, such as CD69 and CD49a, while concomitantly increasing circulatory markers, including CX3CR1, CCR7, CD62L, and S1PR1. In vivo, NKs with RUNX2 silencing displayed a predominant cNK phenotype, with a drastic reduction in the trNK frequency across bone marrow, lungs, spleen, and intestine.

In contrast to the aforementioned TFs governing NK early differentiation, the migratory behaviour of mature NKs was primarily shaped by the activity of **KLF2**. This TF facilitated their homing to interleukin (IL)-15-rich niches, which are essential for NK survival and expansion [22]. It has been suggested that KLF2 expression is crucial for imprinting a homeostatic migration pattern on mature NKs, thereby ensuring access to IL-15-rich environments established by myeloid cells. *Klf2* loss was associated with impaired NK homeostasis, characterized by downregulated expression of CD62L, CX3CR1, and S1PR5, alongside the upregulation of the circulatory marker CCR7. Consequently, the absence of KLF2 triggered aberrant NK migration, restraining their tissue retention and compromising IL-15-mediated recruitment.

#### Tumor recruitment

The primary regulatory axes governing NK cell recruitment to tumors rely on the transforming growth factor beta (TGF-β) signaling and its downstream canonical mediators. A TGF-β-enriched TME upregulated transcripts associated with NK tumor homing (*CXCR3*, *CXCR4*) and tissue residency (*ZNF683*, *ITGAE*) [23]. These effects were partially mediated by SMAD4. Notably, SMAD4 loss rescued the TGF-β-induced tissue-residency features, preventing the downregulation of the integrins CD49b, CD11a, CD11b, and CD49d [23]. However, TGF-β upregulated other tissue-residency markers, such as CD29, CD49a, and CD103, independently of *SMAD4* deletion. SMAD4 exhibited TGF-β-independent signaling that antagonized the regulation of integrins and chemokine receptors. *SMAD4*-deficient NKs displayed an enhanced binding affinity for colon cancer spheroids under homeostatic conditions, and, upon TGF-β treatment, this divergence from control cells was further amplified, resulting in deeper NK infiltration and migration toward a chemokine gradient [23]. A comparable effect was observed in melanoma-bearing mice following the SMAD3 loss, another member of the SMAD family. In this model, *Smad3* depletion was associated with an increased frequency of CD49b^+^ NKs in the blood and spleen, alongside higher recruitment in the TME [24].

An alternative downstream mediator of TGF-β signaling is the zinc-finger TF HOBIT (Homolog of Blimp-1 in T cells, *ZNF683*), which was highly expressed in CD56^bright^ TiNKs in bladder cancer. HOBIT expression correlated with residency markers (CD103, CXCR6) and drove NK recruitment in the TME [25]. Consistently, tissue-egress factors, such as S1PR1 and CCR7, showed a negative correlation with HOBIT levels. This transcriptional profile was also a distinctive feature of human lung trNKs [12], further supporting a conserved role for HOBIT in orchestrating NK tissue residency and tumor infiltration.

**Fig. 1:**
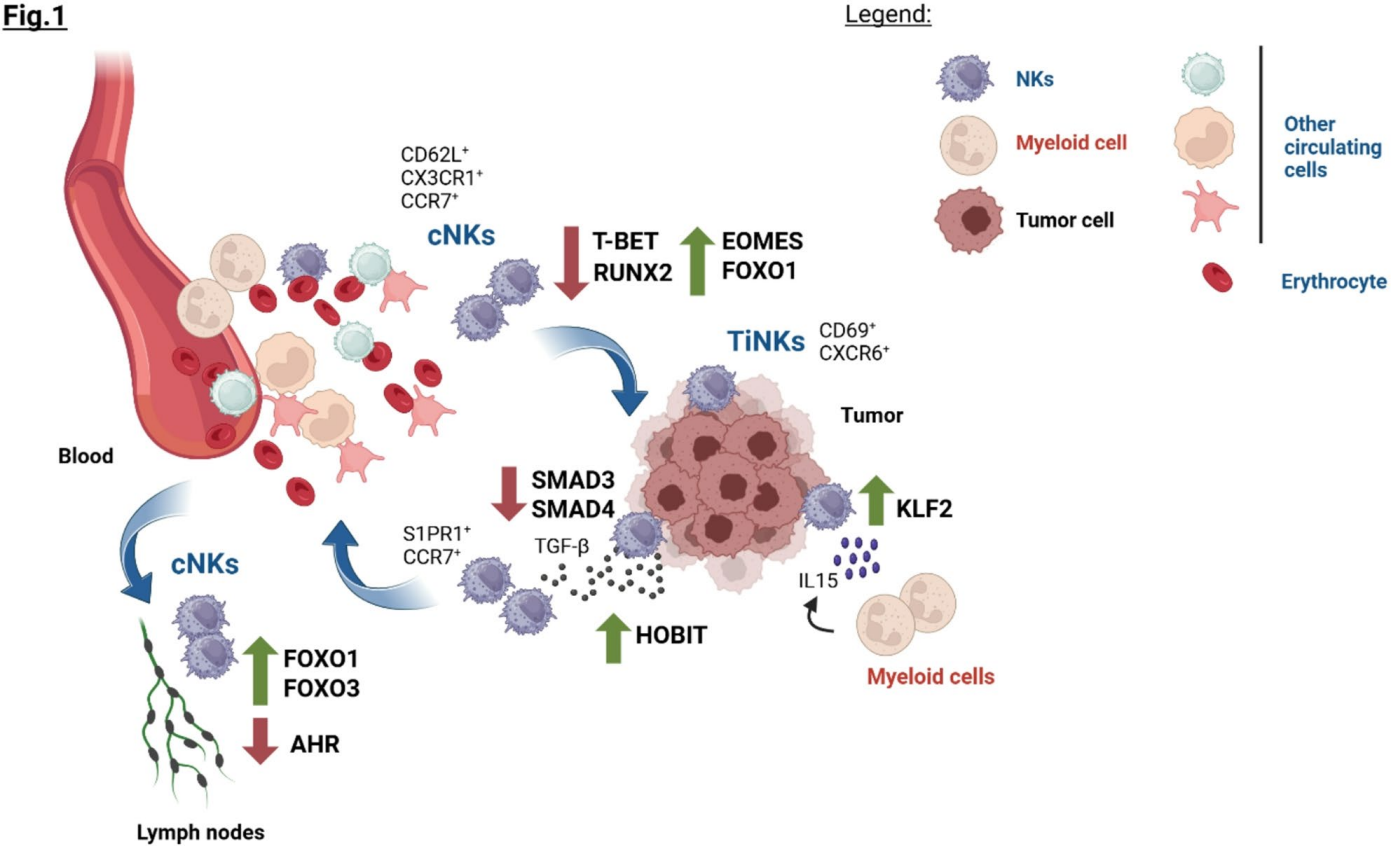
TFs controlling NK homing and tumor recruitment. Schematic overview of NK trafficking and homing governed by the transcriptional activity of distinct TFs. The surface expression of integrins, selectins, and chemokine receptors defines distinct NK phenotypes, including circulating conventional NKs (cNKs), tissue-resident NKs, or tumor-infiltrating NKs (TiNKs). Each one is characterized by a balanced TF expression and activities that are also dependent on the interaction with other cells/signaling pathways within the surrounding tumor ecosystem. Created with Biorender.com

Collectively, NK trafficking and tumor recruitment primarily rely on a coordinated network of distinct TFs that serve as crucial mediators of differentiation and maturation. Beyond these development-associated TFs, the tumor ecosystem can actively rewire the transcriptional landscape of TiNKs, modulating their persistence and egress potential (Fig. 1).

### Transcriptional networks controlling target recognition and NK activation

**Fig. 2:**
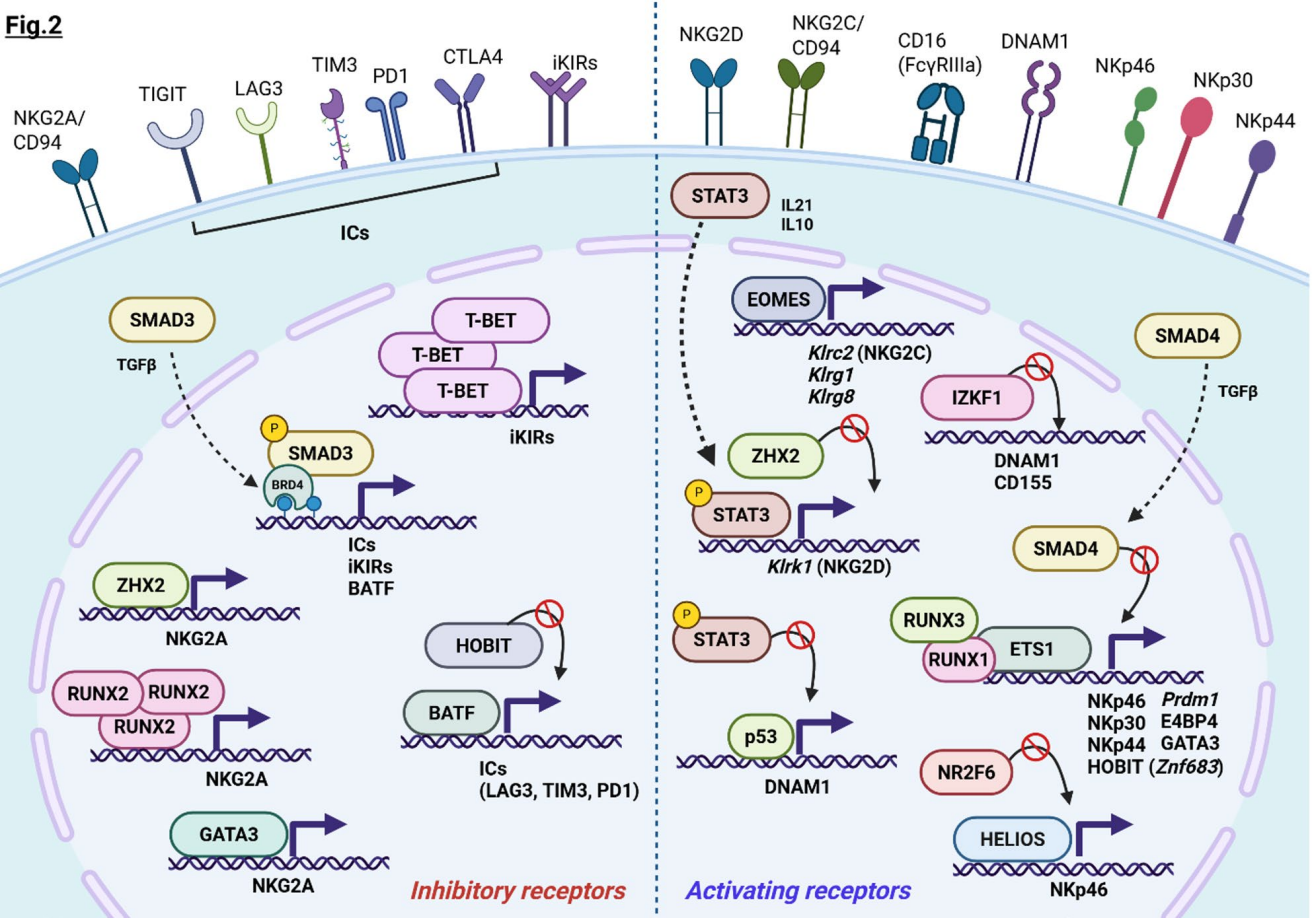
Overview of the transcriptional networks regulating inhibitory/activating surface receptors in NKs. Schematic illustration of the TFs controlling the expression of inhibitory (*left*) and activating receptors (*right*). The large majority of TFs controlled the expression of target genes by promoting transcription initiation, cooperating with other cofactors and TFs. Still, some TFs repress the transcriptional activity of other TFs by interfering with the starting complex recruitment or by inhibiting their expression within a regulatory transcriptional circuit. Created with Biorender.com

NK-mediated immunosurveillance is a dynamic, regulated process, relying on the integration and balance of signals from inhibitory receptors, such as immune checkpoints (ICs), killer cell immunoglobulin-like receptors (KIRs), CD94/NKG2A heterodimers, and activating receptors, including NKp30, NKp46, NKp44, CD16, and NKG2D [26]. Tumors fine-tune ligand expression to remain below the NK activation threshold, thereby facilitating immune evasion. The molecular interface established between NKs and tumor cells, known as immunological synapse, is pivotal for an effective immune response. The assembly of a stable synapse ensures a robust NK engagement with malignant cells, promoting downstream NK effector properties. Notably, the expression and activity of specific TFs can modulate the surface receptor expression, drastically impacting overall NK anti-tumor efficacy (Fig. 2). In this context, TGF-β signaling acts as a major driver of IC upregulation, while, at the same time, serving as the principal inhibitory pathway of activating receptor expression.

#### Inhibitory receptors

A key regulator of NK cell inhibitory receptors is SMAD3, which promoted the expression of ICs and inhibitory KIRs (iKIRs) in NK92 cells and non-small cell lung cancer (NSCLC) TiNKs [27]. Mechanistically, SMAD3 cooperated with the epigenetic reader Bromodomain-containing protein 4 (BRD4), under the sustained influence of TGF-β. ChIP analysis confirmed that SMAD3 bound IC and iKIR promoters in NKs, controlling their expression. Notably, the silencing of either SMAD3 or BRD4 was sufficient to downregulate the expression of the programmed cell death protein 1 (PD-1, *PDCD1*), CD152 (Cytotoxic T-lymphocyte associated protein 4, *CTLA4*), T cell immunoglobulin and mucin domain-containing protein 3 (TIM3, *HAVCR2*), lymphocyte-activation gene 3 (LAG3), T cell immunoreceptor with Ig and ITIM domains (TIGIT), and several iKIRs (KIR2DL1, KIR2DL3, KIR3DL3, and KIR2DL4). This reduction facilitated the formation of a more stable immunological synapse and restored an efficient NK anti-tumor response in ex vivo and preclinical models [27].

Furthermore, in co-cultures with acute myeloid leukemia (AML) cells, SMAD3 induced the expression of BATF (Basic leucine zipper ATF–like transcription factor) by binding to its enhancer upon activation of the canonical TGF-β pathway [28]. ChIP analysis identified multiple ICs as BATF direct targets, and the TF overexpression was associated with increased surface exposure of LAG3, TIM3, and PD-1.

Unlike other inhibitory receptors, the CD94/NKG2A axis was not influenced by SMADs’ direct or indirect regulation. Instead, it was positively regulated by RUNX2 overexpression [21], zinc-fingers and homeoboxes 2 (ZHX2) transcriptional activity [29], and GATA-3 transactivation via direct binding to the NKG2A promoter [30].

Conversely, other TFs act as negative regulators of IC expression. A prominent example is HOBIT, previously noted as a key determinant of NK tumor residency. In bladder cancer, HOBIT restrained the relative abundance of LAG3, TIM3, CTLA-4, and PD-1 on NKs [25]. This inhibitory effect was particularly relevant in CD56^bright^ NKs, supporting its role as a negative master regulator of ICs.

Other development-associated TFs exhibited a distinct role in regulating inhibitory receptors. While EOMES appeared not to be involved, T-BET overexpression increased the expression of iKIRs, including KIR2DL1, KIR2DL3, KIR2DSA, and KIR3DL1, through an augmented chromatin access to their promoters during NK differentiation [31].

#### Activating receptors

Activating receptors are governed by a distinct subset of TFs compared to inhibitory molecules, suggesting a complementary and non-redundant transcriptional network (Fig. 2).

ETS proto-oncogene 1 (ETS1) is the founding member of the ETS family, with an established role in NK maturation and activating receptor expression [32]. *Ets1* deficiency resulted in a decreased expression of the activating receptor NKp46, Ly49 receptors, and their associated signal transduction proteins. These results were further supported in human NKs, where the loss of functional *ETS1* was associated with a drastic reduction in NKp46, NKp44, and NKp30, while NKG2D remained unaffected. These *ETS1*-deficient NKs exhibited impaired activation when co-cultured with K562 tumor cells [33].

ChIP-seq analysis revealed a transcriptional cooperation between ETS1 and RUNX1, characterized by shared binding of adjacent genomic regions. ETS1 typically occupied promoter sequences, while RUNX1 targeted enhancer elements. Notably, both RUNX1 and RUNX3 bound the upstream enhancer element of *NCR1* (NKp46), further substantiating their interplay with ETS1 [34]. *ETS1* depletion was also associated with the downregulation of other key TFs, including BLIMP1 (*PRDM1*), E4BP4, GATA3, and HOBIT, underscoring the hierarchical complexity of NK activation [33]. Conversely, the TF HELIOS, encoded by *IKZF2* and belonging to the IKAROS family, was not influenced by *ETS1* loss [33], constituting an ETS1-independent regulatory axis of NKP46 expression and NK activation [35].

Another member of the family, IKAROS family zinc finger 1 (IKZF1), was recently described as a transcriptional repressor of the activator receptors DNAX accessory molecule 1 (DNAM1). The conditional genetic inactivation of *Ikzf1* in NKs conferred superior cytotoxicity, driven by the increased expression of DNAM1, which recognized its ligand, CD155 (PVR), highly expressed on metastatic melanoma [36].

A potent negative transcriptional repressor of NKp46 is the nuclear orphan receptor subfamily 2 group F member 6 (NR2F6), which showed a direct binding to the *Ncr1* promoter [37]. In germline *Nr2f6*-deficient mice, NKp46 expression was enhanced across bone marrow-derived NKs, cNKs, and TiNKs, resulting in superior immunosurveillance. This effect was associated with a marked reduction in the metastatic spread of MHC-I-deficient melanoma, positioning NR2F6 as a critical negative regulator of NKp46-dependent activation [37].

The NKG2D receptor (*KLRK1*) is regulated by independent transcriptional mechanisms, primarily involving signal transducer and activator of transcription 3 (STAT3) signaling [38, 39]. Stimulation of STAT3 via IL-21 or IL-10 increased NKG2D expression, with phosphorylated STAT3 (pSTAT3) binding directly to the *KLRK1* upstream regulatory region [38]. Notably, STAT3 has also been shown to transcriptionally repress the activating receptor DNAM1 [40], suggesting a context- or stage-dependent regulatory role in NK activation. DNAM1 was also identified as a direct transcriptional target of p53, whose activation may augment NK-mediated immune response in neuroblastoma [41].

Among negative transcriptional regulators, the conditional deletion of ZHX2 enhanced NKG2D expression; when combined with a concomitant reduction in NKG2A levels, this conferred improved lymphoma killing ability in mouse models [29]. Conversely, the TGF-β-enriched TME induced the downregulation of NKG2D, CD16, and NKp30 via SMAD4 signaling, notably without affecting the expression of inhibitory receptors [23].

Finally, a large cluster of NK activating receptors and co-factors was identified as EOMES-specific targets, including *Klra8* (CLEC12A), *Klrg1*, *Klrc2* (NKG2C), and the signaling adaptor protein SAP (*Sh2d1a*) [16]. These findings suggest that EOMES may counteract T-BET activity by favouring a repertoire of activating receptors, thereby improving the overall NK activation potential [16].

#### Cell-death ligands

The expression of cell-death ligands on the NK cell surface represents another pivotal mechanism driving anti-tumor properties. NKs induce apoptosis in target cells through the expression of surface death molecules, including Fas ligand (FasL) and tumor necrosis factor-related apoptosis-inducing ligand (TRAIL). FasL binds to CD95 (*FAS*), while TRAIL engages TRAIL-R1 (*DR4*) and TRAIL-R2 (*DR5*), thereby activating extrinsic apoptotic pathways via their death domains [42].

FasL is the most characterized ligand and is reported to be a shared transcriptional target induced by EOMES and T-BET during NK differentiation [16]. Conversely, FasL expression was negatively regulated by the inducible cAMP early repressor (ICER), which bound the *FASLG* promoter at a site nearly adjacent to the nuclear factor of activated T cells (NFAT) binding region. This transcriptional interplay between NFAT and ICER was shown to repress FasL expression in NKs [43]. Conversely, FasL was positively regulated by the IKAROS factor IKZF1 [36].

While TRAIL expression is restricted to hepatic trNKs and a subset of NKs in homeostatic conditions, it can be rapidly upregulated upon cytokine stimulation [44]. TRAIL-dependent tumor clearance was critical for the control of liver metastases in the presence of IFNγ [44]. Transcriptomic profiling revealed that TRAIL expression in NKs was associated with EOMES absence, but T-BET expression [45]. Furthermore, IL-2 and IL-15 stimulated TRAIL expression through the coordinated interplay between the transcriptional activator NAB2 and the repressor early growth response (EGR)-1 [46].

Collectively, the expression of activating and cell-death ligands is governed in NKs by a specific subset of TFs, separate from those regulating inhibitory and IC receptors. Many of these TFs also act as primary modulators of NK cell homing and differentiation, effectively coupling developmental programs with anti-tumor surveillance and target recognition.

### Regulatory circuits modulating NK effector properties

Beyond their role in shaping the repertoire of NK surface molecules, a complex transcriptional network orchestrates NK effector functions. NKs recognize stressed, senescent, virus-infected, and tumor cells, bypassing the need for prior antigen presentation. Instead, they relied on ‘missing-self’ recognition, which activates NKs against transformed cells that lack or downregulate MHC-I molecules. NKs eliminate these abnormal cells through several cytotoxic mechanisms, primarily the exocytosis of lytic granules. These lysosome-related organelles contain perforin and granzymes, which induce direct target cell lysis in the absence of MHC-I molecules or activator receptor engagement.

Another NK-activating mechanism is antibody-dependent cell-mediated cytotoxicity (ADCC). ADCC is mediated by the bifunctional structure of IgG molecules, which provide a molecular bridge between the tumor-associated antigens and the NK activating FcγRIIIa receptor CD16. Specifically, these molecules are engaged respectively by the Fab and Fc portions of the monoclonal antibody, driving target recognition and the lytic attack.

In addition, NKs secrete several pro-inflammatory cytokines (e.g., IFNγ, TNFα, GM-CSF), and chemokines (e.g., CCL1, CCL2, CCL3, CCL4, CCL5, and CXCL18). These molecules shape the adaptive immunity response and regulate innate functions of myeloid cells [47]. IFNγ, released by activated NKs, is a major determinant of T helper 1 (Th1) and M1 macrophage polarization, thereby sustaining a pro-inflammatory tumor immune microenvironment (TIME). Moreover, NKs enhance recruitment and maturation of dendritic cells (DCs) via GM-CSF and IFNγ release. This promotes IL-12 production by DCs, which, in turn, activate CD8^+^ T cell cytotoxicity. NK-derived GM-CSF is also associated with neutrophil recruitment and, together with IFNγ and TNFα, it promotes neutrophil survival, activation, and the generation of the neutrophil extracellular trap (NET) [48].

**Fig. 3:**
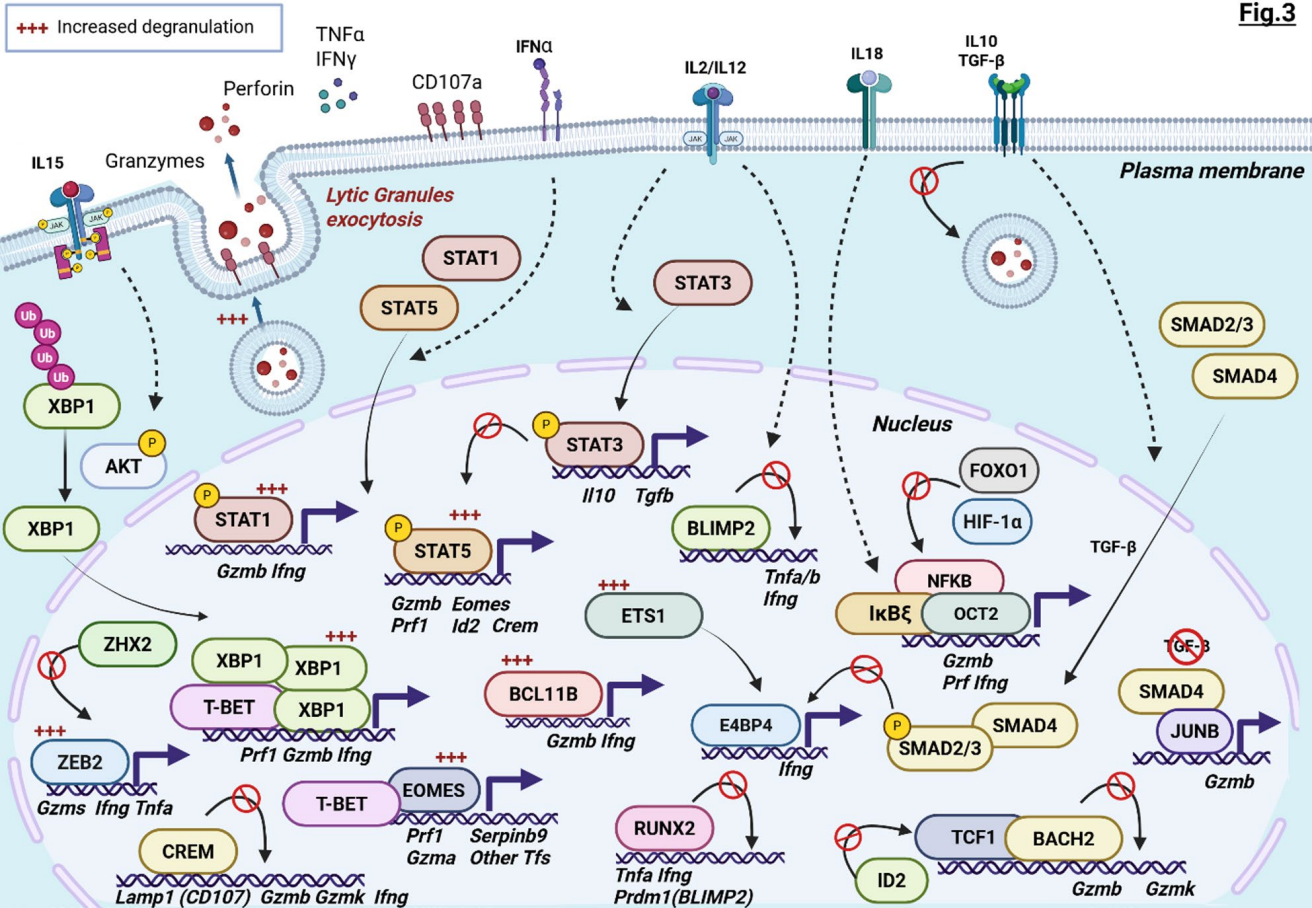
Summary of the positive/negative regulatory mechanisms triggering NK cytolytic activity. Overview of the TF networks that control cytotoxic molecule expression (PRF1, GZMs), the degranulation process (i.e., lytic granule exocytosis associated with surface exposure of CD107a molecules), and cytokine release. Altogether, these TFs define NK anti-tumor potential and prime the surrounding TIME. TF activity is profoundly influenced by cytokine signaling, which determines time- and context-specific mechanisms. Created with Biorender.com

NK cytotoxicity is governed by an orchestrated transcriptional activity of distinct TFs and cofactors that control cytotoxic protein synthesis, lytic granule maturation, and degranulation/exocytosis processes (Fig. 3). Within this hierarchical network, STAT5 and EOMES emerge as master regulators, modulating the expression and activity of subordinate transcriptional circuits.

#### Positive control of NK degranulation

Members of the STAT family are indispensable regulators of NKs, and, among them, STAT1 and STAT5 are key drivers of their cytotoxicity. The role of STAT1 was recently described in IFNα-activated NKs, where its induction increased the expression of granzyme (GZM)B and IFNγ, as well as the release of lytic granules, evidenced by enhanced CD107 surface exposure. This transcriptional program significantly augmented NK killing ability in various tumor models [49]. Similarly, STAT5 stimulated NK proliferation and the production of effector molecules, including perforin (PRF1), GZMA/B, and IFNγ [50]. NKs with diminished levels of STAT5 exhibited a defect in target cell lysis and failed to control leukemia and melanoma progression in mouse models. These findings suggest that, regardless of the NK ability to recognize tumor cells, impaired STAT5 activity inevitably exacerbated tumor growth [50]. Notably, EOMES and ID2 were identified as STAT5 direct transcriptional targets, establishing a clear hierarchy of these factors in regulating NK cytotoxicity [50].

Beyond their established role in NK cell maturation and trafficking, EOMES and T-BET orchestrated specific transcriptional programs driving granule-dependent cytotoxicity. Their co-regulated target genes included *Prf1*, *Gzma*, and *Serpinb9* [16]. Still, T-BET-deficient NKs maintained normal cytotoxicity, whereas EOMES-deficient cells were less cytotoxic, suggesting that EOMES is the hierarchical determinant of NK killing potential [16]. These findings are further supported in an independent study that highlighted EOMES’ superior efficacy in promoting ADCC compared to T-BET [31]. The simultaneous loss of both TFs resulted in impaired IFNγ and TNFα production, alongside decreased degranulation, ultimately leading to a failure in tumor control in leukemia xenografts [51]. Mechanistically, the knockout (KO) of EOMES or T-BET altered the chromatin accessibility at key cytotoxic loci, such as *PRF1* and *GZMM*, underscoring their complementary role in regulating NK cytotoxicity through epigenetic remodeling. The double-deficient model also triggered the dysregulation of other TFs, specifically ZEB2, RUNX3, NFATC2, and KLF2, suggesting a highly coordinated interplay [51].

A putative transcriptional co-operator of T-BET is the X-box binding protein 1 (XBP1), identified as the principal downstream mediator of IL-15-activating signaling pathway [52]. IL-15 stimulated AKT which, in turn, initiated the XBP1 de-ubiquitination pathway, promoting its nuclear translocation. XBP1 overexpression correlated with increased levels of GZMB, IFNγ, and PRF1, alongside a marked expansion of degranulating CD107^+^ cells. NKs carrying XBP1 overexpression exhibited an improved killing ability toward multiple myeloma (MM) cells.

Consistent with the roles of EOMES and T-BET, the overexpression of the zinc finger TF B Cell Lymphoma/Leukemia 11B (BCL11B), known for its established role in NK differentiation, was associated with enhanced production of IFNγ and GZMB, leading to a more robust cytotoxicity toward lung and ovarian carcinoma in vitro and mouse models [53].

Similarly, beyond its role in regulating activating receptors, ETS1 stimulated NK degranulation and IFNγ release upon target recognition. NKs carrying *ETS1*-deficiency exhibited impaired IFNγ release when challenged by leukemia cells [33]. This effect was mediated by one of its direct targets, E4BP4, which occupied the *Ifng* promoter, thereby confirming the ETS1-E4BP4 axis as a critical mediator of the cytotoxic phenotype [24].

Recent single-cell analysis of TiNKs across seven distinct solid tumor types identified a prevalent CD56^dim^ phenotype, characterized by pronounced effector properties. This cytotoxic subset displayed sustained expression of the aforementioned *XBP1* and *TBX21* (T-BET), alongside with other TFs (*ASCL2*, *BATF*, *PRDM1*, *IKZF1*,* KLF2*,* FOXN3*, and *IRF3*) [3]. Such findings underscore the existence of a complex, multifaceted gene-regulatory network that drives the NK cytolytic program in the TME.

#### Negative control of NK degranulation

TFs that negatively regulate NK cytotoxicity outnumber those that directly stimulate the cytolytic phenotype. The primary molecular mechanisms underlying impaired NK degranulation involved the transcriptional repression of cytotoxic molecules, IFNγ, TNF release, and CD107 (*LAMP1*) expression. The activity of these TFs, such as STAT3, SMADs, TCF1, ZHX2, cAMP-responsive element modulator (CREM), and RUNX2, is largely context-dependent and shaped by TGF-β and other cytokines within the surrounding tumor milieu.

STAT3 stands as a primary negative regulator of NK-dependent anti-tumor immunity. ScRNA-seq of TiNKs identified STAT3 as a key determinant of the CD56^bright^ cluster, a subset characterized by impaired cytolytic potential [3]. Distinct studies investigated the absence of STAT3 in NKs. *Stat3*-deficiency resulted in higher release of GZMB, IFNγ, and PRF1 at the steady state, an effect further amplified upon IL-2/IL-12 stimulation, while GZMA remained unaffected [40]. ChIP analysis in primary NKs confirmed IL-12-induced STAT3 binding to the *IFNG* promoter. Notably, the absence of STAT3 enhanced the activation of other members of the STAT family, including pSTAT4 and pSTAT5, which, in turn, triggered lytic molecule production and release. In preclinical melanoma models, STAT3 was constitutively activated in TiNKs [54]. Its ablation led to better tumor control, boosting the cytotoxic ability of spleen-derived NKs following tumor challenge. Beyond direct regulation of cytotoxicity, STAT3 orchestrated a distinct transcriptional program in a subset of CD73^+^ TiNKs, which was described in breast cancer and sarcoma, which served as a major source of the immunosuppressive molecules IL-10 and TGF-β [55]. High levels of these cytokines suppressed CD4^+^ T cell proliferation and activity, thereby facilitating tumor escape [55].

TME-associated immunosuppression is largely driven by TGF-β signaling, which restrains IFNγ production and NK cytotoxicity through the activation of the SMAD family members. SMAD3 promoted tumor immune evasion by upregulating inhibitory receptors [27]. An independent study revealed that the KO of *Smad3* in NKs improved IFNγ production and conferred them a superior capacity to restrain tumor growth in melanoma models [24]. Furthermore, SMAD3 deletion or its pharmacological inhibition led to a marked reduction in angiogenesis, impaired regulatory T cell infiltration, and downregulated the expression of matrix metalloproteinases (MMPs), such as MMP2, MMP9, and MMP13, thereby hindering lung cancer immune escape, invasion, and progression [24]. Mechanistically, TGF-β stimulation increased SMAD3 binding on the nuclear factor interleukin 3 regulated (*Nfil3)* 3’UTR (untranslated region), repressing the expression of E4BP4 protein, which, in turn, was responsible for the IFNγ release [24].

In contrast, the role of SMAD4 in regulating NK cytotoxicity is more uncertain. Its deletion has been associated with failed metastatic spread control in melanoma models, likely due to the decreased GZMB expression [56]. This effect was mediated by the transcriptional cooperation observed between SMAD4 and JUNB on the *Gzmb* promoter [56]. SMAD4 was unable to transactivate the *Gzmb* proximal promoter without JUNB. This positive regulation of SMAD4 appeared to be largely TGF-β-independent. However, this did not preclude the canonical TGF-β-dependent suppression of IFNγ and GZMB, which occurred when SMAD4 cooperated with pSMAD2/pSMAD3 under high TGF-β concentration in the TME. An independent study supported this hypothesis, showing that, upon TGF-β treatment, the levels of GZMB and PRF1 were significantly reduced in human expanded NKs in the presence of SMAD4, but not in SMAD4^KO^ NKs, which maintained their killing ability against colon and breast cancer cells [23]. Collectively, these findings suggest that SMAD4 has context-specific roles in regulating NK cytotoxicity, and this is largely dependent on the relative abundance of TGF-β. At early stages of tumor development, the antitumor role of SMAD4 in NKs may dominate its immunosuppressive functions, leading to an effective immune surveillance. Conversely, at the later stages of cancer progression, abundant TGF-β in the TME likely overwhelms SMAD4’s anti-tumor role, favoring NK immunosuppression.

TGF-β production in the TME is markedly stimulated by the hypoxic environment, a hallmark of solid tumors associated with hypoxia-inducible factor 1 subunit alpha (HIF1α) induction in TiNKs. Notably, the loss of HIF1α in response to the hypoxic TME augmented the intrinsic NK anti-tumor response against MHC-I-deficient lymphoma and lung carcinoma [57]. This enhanced activity was characterized by increased IFNγ, GZMB, and improved NK degranulation. Mechanistically, the anti-tumor phenotype observed in *Hif1a* conditional deleted NKs was mediated by the activation of the nuclear factor kappa-light-chain-enhancer of activated B cells (NFκB)-dependent pathway, which, in turn, switched on IκBξ transcriptional activity and IFNγ production upon IL-18 stimulation. These findings were corroborated by clinical data from cohorts of patients with various solid tumors, including cutaneous melanoma, invasive breast cancer, and cervical carcinoma. An NK-IL-18-IFNγ gene signature derived from TiNKs correlated with patient survival, NK markers, and PRF1 levels, while showing an inverse correlation with HIF1α expression [57]. The cooperation of NFκB with OCT2 and IκBξ was shown to promote NK cytolytic potential upon IL-12/IL-18 stimulation [58]. The activating complex may be counteracted by the negative regulator FOXO1. Its genetic deletion in murine splenic NKs significantly enhanced lytic capacity against a lymphoma cell line, improving IFNγ release upon IL-12/IL-18 stimulation [13].

TCF1 (*Tcf7*) has been identified as a potent negative regulator of GZM expression during NK maturation and tissue residency [59, 60]. It served as a major determinant of the CD56^bright^ phenotype [4, 6], and its expression of TCF1 was positively associated with the repressor BTB domain and CNC homolog 2 (BACH2) [61]. Splenic NKs from *Bach2* conditional KO mice exhibited higher expression and genomic accessibility of cytotoxic effector genes, including *Gzmb* and *Gzmk*. This molecular profile promoted NK terminal maturation and improved anti-tumor control in preclinical models of metastatic melanoma [61]. Furthermore, the *Bach2* conditional deletion elevated GZMB and IFNγ production upon IL-15 stimulation [62]. Mechanistically, TCF1 was significantly downregulated in the absence of BACH2, which explained the observed gain in NK cytotoxicity [61]. TCF1 levels were further negatively regulated by the TF ID2, which controlled the amplitude and temporal dynamics of its expression [63]. NKs carrying *Id2* deletion showed increased chromatin accessibility at the *Tcf7* locus, leading to the upregulation of the TCF1-dependent gene program.

Beyond the activation pathway previously discussed, IL-15-mediated signaling represents another crucial axis. ZHX2 has been recently discovered as a putative transcriptional repressor within this pathway [29]. *Zhx2* deficiency significantly enhanced NK cytotoxicity against Yac-1 lymphoma cells both in vitro and in vivo, an effect driven by increased IFNγ, TNFα, and granzyme production. In hepatocellular carcinoma models, the loss of *Zhx2* similarly led to an increased degranulation, measured by CD107 surface expression, further confirming its inhibitory role.

Integrative analysis of assay for transposase-accessible chromatin sequencing (ATAC-seq) and RNA-sequencing (RNA-seq) data identified ZEB2 as the primary downstream target of ZHX2. Notably, the inhibition of ZEB2 was sufficient to rescue the effect observed in *Zhx2*-deficient NKs, suggesting that ZEB2 is the key effector through which ZHX2 restricts NK maturation and degranulation.

Similar to ZHX2, CREM has been recently identified as a transcriptional repressor of IL-15-mediated stimulation. CREM was markedly induced in NKs via STAT5B activation [64]. Its deletion enhanced NK cytotoxicity against renal carcinoma, breast carcinoma, and Burkitt lymphoma, in both 2D and 3D spheroid models. ChIP-seq analysis identified CREM binding across several genes implicated in NK degranulation (*IFNG*, *LAMP1*, *GZMB*, *GZMK*), as well as in calcium signaling, which governs lytic granule trafficking control.

Furthermore, independent studies identified RUNX2 as a negative transcriptional regulator of NK cytokine production. RUNX2 occupied the *TNFA* and *IFNG* loci, and its silencing was associated with increased cytokine production [21]. Finally, BLIMP1 (*PRDM1*) was linked to the restriction of IFNγ and TNFα/β expression upon IL-2/IL-12 stimulation [65], further expanding the network of factors that dampen NK cytolytic potential.

#### Transcriptional differences with ILC1s and ILC1-like cells in tumors

NKs are members of the ILC family, classified within group 1 alongside ILC1s. These subsets share innate features and TF specific expression, such as T-BET for lineage commitment. However, the differential expression of other development-associated factors, including EOMES and HOBIT, serves as the primary molecular distinction governing their specific development and effector functions [66, 67]. Although NKs and ILC1 coexist within tissues, they exhibit distinct and context-specific functions. Recent evidence also suggested a potential transcriptional plasticity, whereby cNKs transitioned into an ILC1-like phenotype in certain tumors [68, 69].

The transcriptional landscape distinguishing ILC1s and NKs in both healthy tissues and tumors has been further elucidated through single-cell analysis [60]. KLF2 emerged to be required for mature NKs, while remaining dispensable for tissue-resident ILC1s. Conversely, ILC1s were characterized by the sustained expression of HOBIT and ID2. Other key ILC determinants included retinoid-related orphan nuclear receptor α (RORα), BCL11B, AHR, IKZF2, and LITAF. Notably, ILC1s exhibited low or no expression of EOMES compared to NKs.

While the chromatin accessibility profiles of NKs and ILCs were largely shared under homeostatic conditions [70], reflecting a common regulatory circuit that distinguished them from ILC2 or ILC3 populations, the distinctive identities were revealed by differential TF motif enrichment. ILC1-specific accessible regions were enriched for ETS1, IRF4, and ELF1 binding motifs. In contrast, NKs showed a significant enrichment for KLF4, KLF5, MAZ, and SP1 motifs. These findings support the divergent transcriptional programs governing the fate of the two subsets within the ILC1 group.

While IFNγ and granzyme production is a hallmark of both cytotoxic ILC1s and NKs, the transcriptional control of their cytolytic potential appeared to be lineage-specific. In ILC1, the cytotoxicity was primarily governed by HOBIT, although the precise molecular mechanisms remained to be elucidated. It has been hypothesized that HOBIT suppressed TCF1 expression, which, in turn, acted as a negative regulator of GZMB expression [71]. Conversely, HOBIT played a less prominent role in regulating NK cytotoxicity, suggesting that this transcriptional axis is a selective driver of ILC1 effector functions. Supporting these lineage-specific regulations, the TF RORα was suggested as a specific regulator of liver-resident ILC1s [72]. Its conditional deficiency reduced IFNγ release and anti-tumor activity in ILC1s, while exerting a negligible impact on the NK cell compartment.

The TGF-β-depedent transition of NKs into an ILC1-like phenotype has been proposed as a driver mechanism of tumor immune escape [68, 69]. These ILC1-like cells represented an intermediate state between cNKs and ILC1, maintaining the expression of EOMES and CD49b, while acquiring CD49a. Functionally, ILC1‐like cells exhibited lower anti-tumor potential, characterized by impaired IFNγ and granzyme release compared to cNKs [73]. This transition was linked to SMAD4 deficiency in NKs, which induced the acquisition of an ILC1-like gene signature, leading to the observed phenotypic shift [68].

Although the precise classification of NK cells, ILC1s, and ILC1-like cells, especially within the complex landscape of the TME, remains a subject of active debate, the identification of specific driver TFs provides a robust framework for resolving the molecular and phenotypic heterogeneity of these innate lymphoid populations.

### Transcriptional factors regulating NK dysfunctional states

**Fig. 4:**
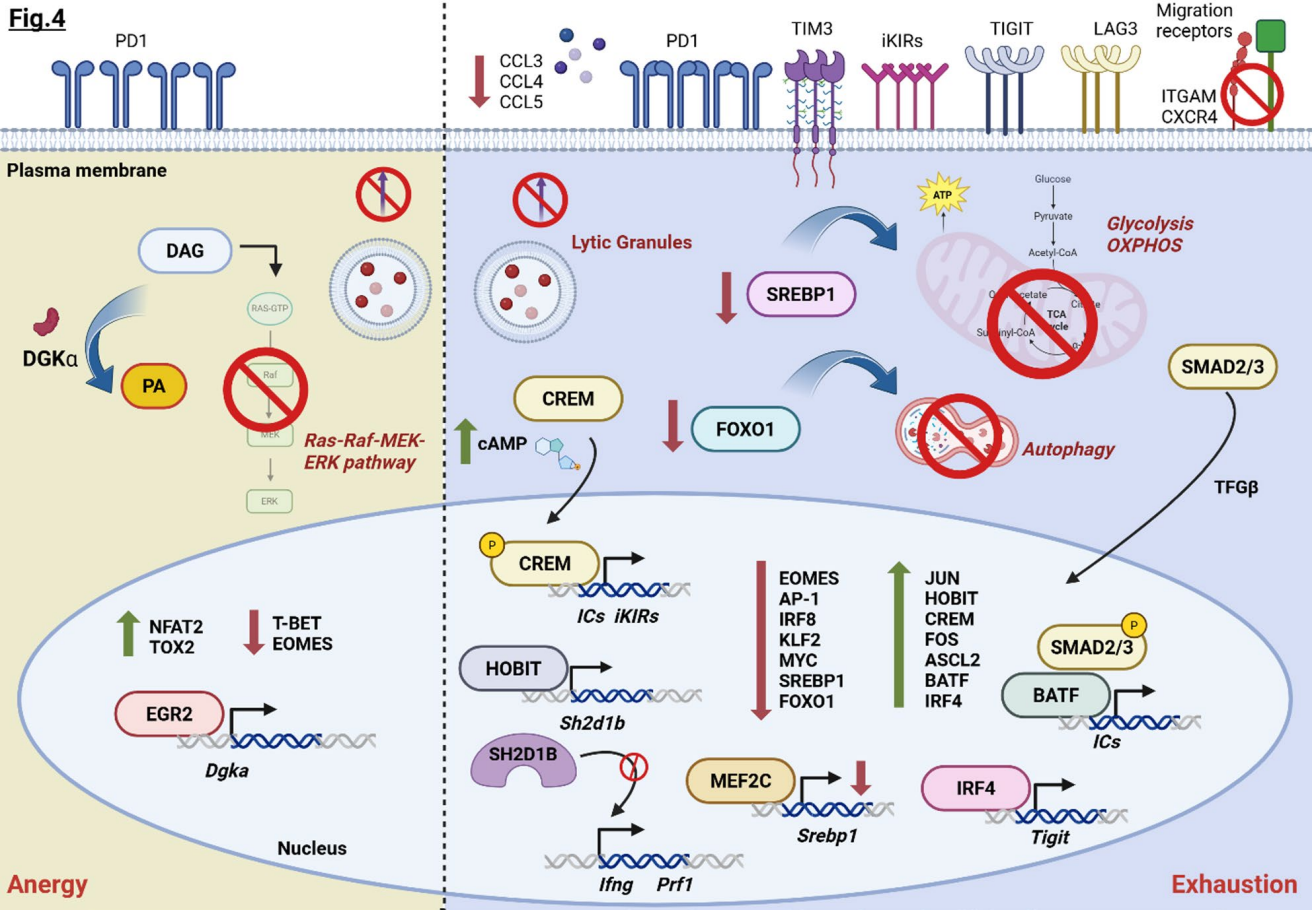
Summary of the transcriptional mechanisms driving NK dysfunctions in TME. Schematic illustration of the distinct molecular mechanisms that characterize dysfunctional NKs in the TME. Different TFs drive NK cells into an anergic state (*left*) or an exhaustion phenotype (*right*). In anergic NKs, the best-known mechanism relies on EGR2 control of DGKα expression [74]. This kinase is responsible for diacylglycerol (DAG) phosphorylation into phosphatidic acid (PA), thus inhibiting the DAG-mediated Ras-Raf-MEK-ERK signaling pathway. scRNA-seq analyses provided a comprehensive transcriptional profile of exhausted NKs, shedding light on the up- or downregulated TFs [88, 91]. Created with Biorender.com

Immersed within the intricate TME, NKs encounter a milieu of immunosuppressive signals, metabolic stress, and cellular interactions that drive them into a dysfunctional state, manifested as either anergy or exhaustion. While anergy and exhaustion may reflect distinct molecular mechanisms of dysfunction, both states are characterized by a relevant decrease in effector functions, including impaired degranulation and lytic potential, alongside restrained proliferation [74] (Fig. 4).

#### Characteristics of dysfunctional NKs

The term “anergy” refers to an intrinsic state of immune functional impairment, applicable to both T and NK cells. In this state, NKs exhibit higher self-tolerance, resulting in generalized hypo-responsiveness toward non-self targets [75, 76]. This phenomenon can arise from chronic exposure to activating ligands in the absence of proper MHC-I inhibitory receptor signaling [76, 77]. Alternatively, within the TME, an anergic state may result from imbalanced inhibitory receptor signaling that destabilizes the immunological synapse, thereby preventing NK activation and facilitating tumor immune escape [78].

Anergic NKs lack inhibitory surface markers such as NKG2A or iKIRs, and express low levels of TIM3 and TIGIT. Conversely, they exhibit sustained PD-1 expression alongside the downregulation of activating receptors [74]. A three-sequential signal model was proposed for NK activation, similar to T cells [79]. Anergic NKs may emerge if one of these signals is missing, such as the co-stimulation of the CD137 activating receptor provided by antigen-presenting cells.

In murine models of MHC-I-deficient tumors, the induction of anergic NKs occurred during the early stages of tumor development [80]. These dysfunctional cells were hypo-responsive to stimulatory antibodies and exhibited defective signal transduction. Notably, while anergic and unlicensed NKs share functional similarities, they relied on different molecular mechanisms. Unlicensed NKs showed hypo-responsiveness and tolerance primarily due to the constitutional absence of inhibitory receptors for MHC-I molecules [81]. In contrast, anergic cells relied on a reduced intracellular signalling transduction upon the engagement of activating receptors. This effect was specifically induced by tumor cells, restricted to the TME and local draining lymph nodes [80]. Furthermore, anergic NKs failed to show an evident causal association with an immature NK subset, differential expression of dominant inhibitory receptors, impaired proliferation, or the concomitant presence of inhibitory cells or cytokines in the TME [80].

The existence of an NK exhaustion phenotype in the TME was first described by Gills and colleagues, highlighting functional similarities with exhausted CD8^+^ T cells [82]. The term “exhaustion” refers to a state of desensitization resulting from NK overactivation upon persistent stimulation, and subsequent failure in target clearance. This dysfunctional phenotype drastically reduces NK cytotoxicity and further prevents interactions with other tumor cells. Exhaustion is characterized by restrained effector functions, the upregulation of inhibitory receptors, including PD-1, TIGIT, TIM3, LAG3, NKG2A, CD96, and a concomitant downregulation of activating receptors, such as NKG2D, CD16, DNAM1, NKp30, and NKp44 [83]. While proficient NK activation requires sustained glycolysis and oxidative phosphorylation (OXPHOS), and increased expression of nutrient transporters, enzymes, mitochondrial mass, and metabolic fluxes [84], exhausted NKs exhibited a general loss of metabolic fitness [85]. This metabolic decline severely restrained the anti-tumor efficacy of adoptive NK-based cell therapies.

Despite overlapping characteristics and phenotypes, anergy and exhaustion are governed by distinct transcriptional signatures [74]. However, the precise definition and molecular boundaries of these dysfunctional states remain partially controversial and have yet to be fully established [86, 87]. Further studies are required to elucidate the etiology and the broader spectrum of NK hypo-responsiveness in the context of tumors.

#### Anergy

The zinc-finger TF EGR2 has been identified in NKs as a pivotal transcriptional regulator of diacylglycerol kinase alpha (DGKα), a key factor associated with NK anergy. The EGR2-dependent expression profile mirrored the transcriptome of exhausted T cells, suggesting a conserved molecular program and shared upstream regulators across different lineages. Mechanistically, EGR2 silencing rescued NK cytotoxic potential, decreasing PD-1 expression both in vitro and in preclinical models [74]. Furthermore, the same study associated the anergic phenotype with reduced expression of EOMES and T-BET when compared to activated NKs.

The genomic profile of anergic NKs largely overlapped with signatures related to NFAT and thymocyte selection associated high mobility group box (TOX). This suggests that the upregulation of NFAT2 (*NFATC1*) and TOX2 may be critical in defining this dysfunctional state [74]. However, to date, functional studies validating the role of NFAT2 and TOX2 as drivers of NK cell anergy remain unavailable.

#### Exhaustion

In preclinical AML models, NK exhaustion during adoptive cell transfer has been largely associated with the progressive loss of both EOMES and T-BET expression. Notably, only the rescue of EOMES was sufficient to prevent exhaustion, suggesting a more central role of the TF in restraining NK dysfunction during prolonged TME exposure [82]. In trNKs, EOMES expression is lost in favor of T-BET [16], and this molecular switch, while crucial in the establishment of NK tissue residency, may subsequently predispose cells to a dysfunctional phenotype upon chronic stimulation.

To further elucidate the dynamic fate of NKs within the TME, single-cell temporal labeling was employed to map cell progression towards a terminal exhausted pseudotime [88]. This analysis revealed a relevant upregulation of inhibitory receptors and decreased cytotoxicity. Furthermore, a marked reduction in migration-associated transcripts (*Cxcr4* and *Itgam*) and chemokines (CCL3, CCL4, and CCL5) was observed, potentially influencing dendritic cell recruitment. SCENIC gene regulatory network analysis predicted the drivers of this transition, identifying AP-1 family members, interferon regulatory factor (IRF)-8, KLF2, and MYC, as being downregulated in exhausted NKs. Additional functional studies will be required to confirm the role of these TFs in preventing NK exhaustion in the TME.

Among these TFs, a functional validation was recently provided for KLF2. Previously, the factor has been described as a primary driver of NK cell migration toward IL-15 niches and a key determinant of tissue residency [22]. Maintaining KLF2 expression in trNKs was demonstrated to be critical for the immune response under chronic stimulation in the TME [89]. The rescue of KLF2 expression in trNKs enhanced their cytotoxicity and prevented the acquisition of an exhausted, dysfunctional state.

Single-cell temporal labelling of exhausted NKs revealed no significantly upregulated TFs [88], consistent with previous findings on EOMES loss [82]. This suggests that exhaustion primarily relies on the reduced activity of core TFs essential for NK activation and expansion.

In contrast with this hypothesis, JUN was identified as a critical driver of NK exhaustion in T cell acute lymphoblastic leukemia (T-ALL) [90], where it triggered IC expression, suggesting a temporal and context-specific role.

Similarly, HOBIT (*ZNF683)* has been implicated in driving NK exhaustion within MM [91]. ScRNA-seq of MM patients identified a HOBIT-expressing NK cluster with a marked exhausted phenotype. Notably, HOBIT KO effectively rescued NK cytotoxicity and reduced inhibitory receptor expression [91]. Mechanistically, HOBIT occupied the *SH2D1B* gene promoter, downregulating its expression. The impaired expression of this adaptor protein led to a drastic reduction in IFNγ and PRF1 synthesis, a hallmark of NK exhausted phenotype in the bone marrow TME [91]. HOBIT has been shown to restrain the development of IFNγ-expressing NKs [92] and was constitutively highly expressed in CD56^bright^ trNKs in bladder cancer [25] and liver tissues [93].

Conversely, the exhausted NK cluster identified in MM by Li et al. [91] showed a CD56^dim^-associated transcriptional signature. The expression dynamics of HOBIT coincided with the transition from the immature CD56^bright^ NK cluster to the exhausted population, in which HOBIT levels were upregulated. These findings suggest that HOBIT-mediated NK exhaustion and the subsequent impairment of cytolytic activity in MM operate independently of its established role as a tissue resident determinant in CD56^bright^ NKs.

Among recent studies aiming to identify NK exhaustion master regulators, CREM has been implicated in the dysfunctional phenotype observed in infused CAR-NKs [64]. Transcriptomic analysis of CAR19-IL-15 NKs in a murine lymphoma model revealed that CREM, FOS, and ASCL2 were upregulated compared to the steady state. Notably, this study highlighted for the first time the role of CREM in NKs. Higher CREM expression in infused CAR-NKs correlated with improved tumor control in vivo, yet was accompanied by a concomitant increase in inhibitory markers, further supporting the link between CREM and activation-induced exhaustion [64]. Similarly, TiNKs, particularly those from bladder cancer, cholangiocarcinoma, and lung cancer, exhibited elevated CREM expression, which was associated with a more mature phenotype. An independent study confirmed that CREM transcriptional activation characterized dysfunctional NKs in AML. In this context, AML blasts secreted high levels of prostaglandin E2 (PGE2), a potent inducer of cAMP that, in turn, activated CREM [94].

BATF has been associated with AML-induced exhaustion, being directly stimulated by TGF-β and SMAD2/3 activation [28]. Sustained transcriptional activity of BATF increased IC expression, while its deletion rescued NK effector properties against tumor cells.

Similarly, in the context of metastatic melanoma, IRF4 has emerged as a key mediator of NK exhaustion. Its loss reversed murine NK exhaustion, enhancing their cytotoxic potential and drastically reducing TIGIT expression [95].

While context-specificity remains fundamental in defining the transcriptional signature of NK exhaustion, shared epigenetic remodelling has been reported across the promoter regions of key TFs, including *FOXO1*, *FOXP1*, *SATB1*, *TCF12*, *IKZF3*, *NFATC*, *TOX*, and *ZBTB38* [96]. This common chromatin landscape was observed in both exhausted CD8^+^ T cells and adaptive NKs, unveiling a conserved transcriptional coordination that may drive the exhaustion of cytotoxic immune cells. Additional functional studies are required to validate the suggested transcriptional networks and the associated epigenetic remodelling in both T cells and NKs.

To prevent NK exhaustion, maintaining metabolic fitness is essential to sustain effector functions and a persistent anti-tumor immune response. Upon cytokine stimulation, the activation of glucose metabolism via glycolysis fuels the biosynthesis of amino acids and fatty acids [97]. This metabolic shift was driven by the increased transcriptional activity of the master regulator of lipid synthesis, sterol regulatory element-binding protein 1 (SREBP1), which inhibition caused a defective glycolysis and OXPHOS [97]. Specifically, IL-12/IL-15/IL-18-stimulated NKs subjected to pharmacological SREBP1 inhibition failed to upregulate IFNγ and GZMB, showing less cytotoxicity against lymphoma cells. SBREPB1 was, in turn, transcriptionally controlled by myocyte-specific enhancer factor 2 (MEF2C), which was induced during IL-2/IL-15 stimulation, identifying it as a key upstream driver of SREBP1-mediated lipid metabolism [98].

Interestingly, murine TiNKs from breast carcinoma and melanoma models displayed a unique enrichment of glycolysis-associated genes, including *Aldoa*, *Tpi1*, *Pgk1*, *Pkm2*, and *Ldha*, indicating a sustained hypoxia-driven response [60]. Compared to trNKs or cNKs, TiNKs showed differential expression of key TFs that likely contributed to this glycolytic phenotype, including IRF8 induction or KLF2 downregulation. These findings are supported by independent data identifying a CD56^bright^ human TiNK cluster with a stressed/exhausted phenotype [3]. These cells exhibited increased hypoxia-driven gene expression and enhanced glycolytic metabolism, alongside high expression of IRF8 and negligible KLF2 levels [3]. While the functional rescue of KLF2 has been shown to prevent NK cell dysfunction within the TME [89], further validation is required to confirm whether IRF8 acts as a driver of NK metabolic exhaustion.

TiNKs were characterized by dysfunctional autophagy resulting from exposure to altered TME, which was associated with abnormal mitochondria polarization, impaired effector functions, and an exhaustion-like phenotype [99]. FOXO1 was identified as the main transcriptional regulator orchestrating NK autophagy by controlling the expression of the autophagy-related protein 7 (ATG7) [100]. Mechanistically, the cytosolic phosphorylated form of FOXO1 interacted with ATG7 within autophagosomes, stimulating the autophagic process. The loss of either *Foxo1* or *Atg7* abrogated autophagy in NKs, drastically restraining their effector functions. Notably, FOXO1 governs this transcriptional program independently of its established role in NK homing and the negative regulation of terminal differentiation, which were instead mediated by the downregulation of its downstream target T-BET [18].

Overall, dysfunctional NKs are characterized by distinct molecular mechanisms that restrain their effector properties, enhance susceptibility to tumor-immune escape, and drive immune tolerance. Beyond the well-established regulation of surface receptors, ICs, and cytotoxic molecules, NK dysfunction is fine-tuned by metabolic dynamics that dictate their persistent long-term efficacy within the tumor ecosystem (Fig. 4).

### Exploiting TF targeting as a novel therapeutic opportunity

NK-based cell therapies against hematological malignancies have demonstrated both safety and efficacy in clinical settings [1, 2]. However, enhancing NK cell potency and persistence remain unmet clinical need for the optimization of cell therapies, especially in solid tumors.

The identification of novel TF-dependent regulatory axes that potentiate the in vivo anti-tumor killing ability of NKs is essential for the clinical advancement of NK-based therapies.

The modulation of TF expression in NKs, achieved through pharmacological targeting or a genome editing approach, offers a strategy to simultaneously enhance different NK anti-tumor properties. These include improvement of TME recruitment and persistence, enhanced cell cytotoxicity, and the prevention of dysfunctional states, thereby expanding the therapeutic applicability of NK cell-based immunotherapies (Table 1).

**Table 1:** List of the main strategies targeting TF to improve NK anti-tumor response. Overview of the pharmacological or genome editing-based preclinical approaches to modulate the activity of different TFs in NKs. For each approach, the strategy, the relevant model system, and tumor context are listed, together with the main findings/outcomes and the relative reference

Target TF	Strategy	Compound	Tumor context	Model system	Observed Effect/Outcome	Reference
AHR	Small-molecule agonist	FICZ	OSCC	Syngenic mouse models (MOC2); xenograft mouse models (SSC-4, UM-SCC-103)	Increased migration and tumor infiltration of adoptive NKs.	[20]
BATF	Genome Editing (CRISPR/Cas9)	/	AML	Xenograft mouse models (THP-1, OCI-AML3)	Reduced tumor growth induced by adoptive NKs carrying BATF KO. Impaired NK exhaustion.	[28]
BCL11B	Genome editing (CRISPR/Cas9 knock-in)	/	OC	Xenograft mouse models (OVCAR8)	Reduced tumor growth and increased mouse survival with adopted iPSC-derived NKs carrying BCL11B overexpression. Increased NK cytotoxicity with higher expression of PRF1 and GZMB.	[53]
BLIMP-1, T-BET, ZEB2	Small-molecule inhibitor	CHIR99021 (GSK3 inhibitor)	OC	Xenograft tumor models (SKOV-3)	Reduced tumor growth when adoptive NKs were ex vivo pre-treated with the inhibitor. Increased anti-tumor efficacy in combination with Trastuzumab.	[102]
CREM	Small-molecule inhibitors	Celecoxib and EP2/4 antagonists (PGE_2_ inhibitors)	AML	Ex vivo co-cultures between patient-derived NKs and AML blasts	Prevented tumor-induced NK inhibition. Rescued IFNγ and TNFα release, increased NK degranulation, and NKG2D expression.	[94]
	Genome editing (CRISPR/Cas9)	/	BC, Burkitt Lym, PDAC	PDX (BCX.010, PATC148); Xenograft mouse models (Raji)	Enhanced CAR-NKs efficacy with reduced tumor growth and increased mouse survival. In BC, decreased metastatic spread. Increased CAR-NK persistence and cytotoxicity.	[64]
EGR2	Short interference RNA (siRNA)	Lipid-based nanoparticles	PDAC	Xenograft mouse models (PANC-1)	Rescued NK exhaustion, increased anti-tumor efficacy, and higher tumor control. Reduced IC expression (PD-1) and increased NK degranulation.	[74]
EOMES	Genome editing (Overexpression with retroviral vector)	/	B cell Lym	Syngenic mouse models (A20)	Tumor growth reduction and increased mouse survival with adapted NKs overexpressing EOMES.	[82]
GATA3	Small-molecule inhibitors	Dasatinib, Imatinib, Nilotinib (TKIs)	CML	Ex vivo analysis of TKI-treated patient-derived NKs and co-cultures with K562 cells	Increased expression of NK activating receptors (NKG2D, NKp30, DNAM1) and reduced expression of inhibitory receptors, NKG2A and KIR3DL1 (Dasatinib only). In co-cultures, enhanced NK degranulation.	[103]
HIF1α	Small-molecule inhibitor	KC7F2	CML	Ex vivo expanded hNKs in co-culture with K562 cells	Enhanced NK degranulation with increased release of IFNγ, TNFα.	[57]
	Genome editing (Cre-LoxP)	/	NSCLC, T cell Lym	Syngenic mouse models (LLC1, RMA-S)	Reduced tumor growth in mice with endogenous or adoptive NKs carrying *Hif1a* conditional deletion. Increased NK activation with higher IFNγ and GZMB production.	[57]
IRF4	Genome editing (Cre-LoxP)	/	Mel	Syngenic mouse models (B16-F10)	Reduced NK exhaustion and IC expression (TIGIT), with increased production of IFNγ and PRF1.	[95]
JUN	Small-molecule inhibitor	JKN-IN-8	T-ALL	Syngenic mouse models (NOTCH1-mutant T-ALL)	Reduced tumor growth and extended mouse survival. Increased NKs in the bone marrow and prevented their exhaustion by impairing IC expression (PD-1, TIGIT, LAG3). The effect was higher in combination with chemotherapy and IC blockade.	[90]
P53	Small-molecule inhibitor	Nutlin-3a	NB	Xenograft mouse models (LA-N-5); ex vivo co-cultures of patient-derived tumor spheroids and hNKs	Reduced in vivo tumor growth with enhanced mouse survival and increased adoptive NK tumor recruitment. In co-cultures, enhanced spheroid shrinkage and tumor-induced apoptosis.	[41]
SMAD3	Small-molecule inhibitor	SIS3	Mel, NSCLC	Syngenic mouse models (B16F10, LLC1)	Enhanced NK differentiation; Reduced tumor growth and increased mouse survival; Increased NK tumor recruitment and release of GZMB, IFNγ, and IL-2 in the TME and circulation.	[24]
	Epigenetic Drugs	JQ1, OTX015 (BET inhibitors)	NSCLC	Xenograft mouse models (NCI-H23; NCI-H1299); Syngenic mouse models (LLC1); ex vivo co-cultures of patient-derived tumor spheroids and TiNKs	In xenografts with adoptive transfer of NK92 cells, reduced tumor growth. In syngenic models, potentiated endogenous NK anti-tumor effects by reducing ICs and enhancing degranulation. In co-cultures, enhanced spheroid shrinkage and rescued TiNK degranulation.	[27]
SMAD4	Genome Editing (CRISPR/Cas9)	/	BC, CRC	Xenograft mouse models (HCC1954, HCT116)	Reduced tumor growth induced by adoptive NKs carrying SMAD4 KO. In BC, improved anti-tumor response in combination with Trastuzumab/Pertuzumab.	[23]
SREBP1	Cytokine stimulation	IL-12, IL-15, IL-18	Mel	Syngenic mouse models (B16)	SREBP1 is required to obtain a sustained anti-tumor efficacy of adoptively transferred cytokine-stimulated mNKs and IFNγ/GZMB release.	[97]
STAT3	Small-molecule inhibitor	CPA-7	Bladder Cancer, Mel	Syngenic mouse models (MB39, B16 cells); in vitro co-cultures of mNKs from tumor-bearing mice with YAC-1 cells	Reduced in vivo tumor growth due to T cell and NK cell engagement. Increased NK cytotoxicity within in vitro co-cultures.	[54]
ZHX2	Genome editing (Cre-LoxP) or RNA interference (shRNA)	/	HCC, Mel	Syngenic mouse models (Hepa1-6; B16-F10); Xenograft mouse models (HepG2, Huh7)	Reduced tumor growth, metastases, and increased mouse survival, with adopted NKs carrying ZHX2 deletion or silencing. Enhanced NK tumor recruitment and degranulation.	[29]

*Abbreviations: AML* Acute myeloid leukemia, *BC* Breast cancer, *CML* Chronic myeloid leukemia, *CRC* Colon rectal carcinoma, *HCC* Hepatocellular carcinoma, *Lym* Lymphoma, *Mel* Melanoma, *NB* Neuroblastoma, *NSCLC* Non-small cell lung cancer, *OC* Ovarian cancer, *OSCC* Oral squamous cell carcinoma, *PDAC* Pancreatic ductal adenocarcinoma, *T-ALL* T cell acute lymphoblastic leukemia, *IC* immune checkpoint, *TKI* Tyrosine-kinase inhibitor, *NK* Natural killer, *shRNA* short interference RNA, *IFNγ* Interferon gamma, *GZMB* GranzymeB, *TNFα* Tumor necrosis factor alpha

#### Pharmacological targeting

Historically, TFs were largely considered undruggable targets, due to the limited availability of binding pockets for conventional small-molecule inhibitors [101]. TF functions are often mediated through protein–protein or protein–DNA interactions, which often display featureless interfaces that lack the structural depth for the design of specific inhibitors. These limitations have posed significant structural and technical challenges to the development of potent and selective direct TF inhibitors.

Indirect modulation strategies have been proposed as an alternative option, such as targeting co-factors or manipulating upstream signalling. However, these approaches often suffer from poor specificity and off-target effects, which currently limit their clinical translation.

In the context of NKs, several TFs have emerged as putative targets for direct pharmacological inhibition. STAT3, one of the main negative regulators of NK effector functions [38, 40, 54], was successfully targeted by a small-molecule inhibitor, CPA-7, that was applied to limit melanoma growth, metastatic spread, and potentiate the overall immune response [54]. However, the observed therapeutic effect was only partially dependent on NK activation, relying also on T cell effector functions.

Similarly, the specific SMAD3 inhibitor, SIS3, was developed to block its activating phosphorylation, thereby mitigating TGF-β-dependent NK immunosuppression and restraining lung cancer and melanoma growth in preclinical models [24]. Notably, NK cell depletion only partially abrogated SIS3 anti-tumor effects, suggesting the involvement of other cell players in the TME.

Another example is KC7F2, a pharmacological inhibitor of HIF1α translation, which potentiated NK effector functions by neutralizing HIF1α’s immunosuppressive role. This intervention successfully restored IFNγ production under hypoxic conditions and significantly enhanced NK-mediated cytotoxicity against K562 cells [57].

Alternatively, other drugs can be employed to stimulate TF activity. For instance, administration of the AHR agonist FICZ has been shown to restore the anti-tumor potential of CD56^bright^ NKs by increasing activating receptors, CD69 expression, while downregulating CD62L, and enhancing NK migratory capacity [19, 20]. Similarly, the small-molecule antagonist Nutlin-3a disrupted the inhibitory interaction of MDM2 with p53 [41]. The drug increased p53-mediated expression of NKG2D activating receptor and NK cytotoxicity against neuroblastoma. In xenograft models, combining adoptive NK cell transfer with Nutlin-3a treatment led to significant tumor shrinkage and improved overall survival, positioning Nutlin-3a as a promising candidate for NK-based immunotherapies [41].

Besides the recent advancements in the design of TF-specific drugs, several TFs remain poorly druggable. Consequently, their activity is often indirectly modulated by targeting upstream regulators or downstream transcriptional targets.

A notable example is the selective inhibitor of glycogen synthase kinase 3 (GSK3), CHIR99021, which increased the activity of T-BET, ZEB2, and BLIMP-1, key drivers of NK immunosurveillance [102]. The drug improved NK cytotoxicity, without affecting NK cell viability or proliferation. Ex vivo expanded NKs treated with CHIR99021 and co-cultured with tumor cells exhibited higher production of TNF, IFNγ, PRF, GZMB, activating receptors (NKG2D, DNAM1, NKP30), and enhanced ADCC. The adoptive transfer of CHIR99021-treated NKs displayed a superior tumor control in a xenograft model of ovarian cancer [102].

Another example is SMAD3, whose expression was driven by the bromodomain and extra-terminal domain (BET) protein BRD4. Specifically, BRD4 bound to the *SMAD3* promoter in NKs, thereby mediating its transcriptional activation [27]. BET inhibitors (BETi) belong to epigenetic drugs and block the enzymatic subunit of BET proteins, causing their chromatin detachment. In NSCLC patients, BETi drastically reduced SMAD3 expression in NK92 and TiNKs, thereby improving their overall anti-tumor potential. In mouse models, the superior anti-tumor activity of BETi-treated NKs restrained orthotopic and metastatic tumor growth [27].

Tyrosine kinase inhibitors (TKIs), such as dasatinib, are standard-of-care treatments for chronic myeloid leukemia. TKIs inhibited the p38 mitogen-activated protein kinase (MAPK) pathway, which promoted GATA3 nuclear translocation and activation in NKs [103]. Given that GATA3 regulated the expression of NKG2A inhibitory receptor [30], the TKI-induced enhancement of NK cytotoxicity appeared to be mechanistically linked to the suppression of the p38-GATA3 signaling [103].

Novel therapeutic strategies have been developed to specifically reverse NK exhaustion in the TME. Lipid-based labeled nanoparticles targeting NKp46^+^ NKs were employed to deliver short-interference RNAs (siRNAs) against EGR2 in murine models of pancreatic cancer [74]. This target delivery system minimized off-target effects on non-NK populations and successfully rescued the exhaustion-driven phenotype mediated by EGR2 in TiNKs. NK degranulation was enhanced, leading to an overall reduction of tumor growth.

In the context of T-ALL, an independent study restrained NK exhaustion through JUN inhibitors, either alone or in combination with chemotherapy [90]. The JUN inhibitor JNK-IN-8 decreased tumor growth and prolonged mouse survival. The combination with chemotherapy further enhanced overall anti-tumor response and restrained IC expression. Notably, the combination with IC blockade exerted a synergistic effect in reducing tumor growth, further amplifying NK-mediated immunity.

In an independent study, CREM was indirectly targeted through the pharmacological inhibition of prostaglandin-induced signaling, a pathway known to suppress NK cell function in AML [94]. Treatment with Celecoxib (a COX2 inhibitor) or EP2/EP4 antagonists effectively restored NK cytotoxicity. Mechanistically, these drugs prevented cAMP accumulation and restrained CREM activation in dysfunctional NKs.

The proper activation of SBREP1 was required following cytokine induction to maximize NK anti-tumor response and prevent the acquisition of a dysfunctional phenotype [97]. The pharmacological inhibition of SBREP1, using either 25-hydroxycholesterol (25-HC) or PF-429,242, decreased IFNγ and GZMB production, leading to a general impairment of in vitro cytotoxicity against lymphoma cells [97]. Furthermore, in a syngeneic murine melanoma model, SREBP1 pharmacological inhibition restrained the ability of adoptively transferred NKs to control tumor burden [97]. These findings underscore the critical role of SREBP1-driven metabolic reprogramming in cytokine-stimulated NKs within the TME.

Overall, the reported evidence has been primarily derived from preclinical models (summarized in Table 1). Robust clinical validation remains necessary to assess the feasibility, safety, and therapeutic efficacy of TF pharmacological targeting in endogenous NKs from cancer patients.

#### Adoptive NK-based cell therapies

Several studies suggest that TF modulation represents a novel strategy to maximize NK immunosurveillance in adoptive cell therapies for both haematological and solid tumors. However, TF genome editing remains technically challenging in NKs [104]. Gene editing efficiency is generally restrained by the inherent difficulty of their engineering. Furthermore, engineered NKs often exhibit high susceptibility to apoptosis during manipulation and limited persistence in vivo.

One of the most common targets is the TGF-β-dependent signaling, aiming to prevent the effect of the immunosuppressive TME on adopted NKs. *SMAD4*-deficient NKs demonstrated superior control of breast cancer growth, both as single-agent therapy or in combination with anti-HER2 antibodies [23]. In expanded human NKs, SMAD4 loss did not impact cell cytotoxicity or cytokine production in homeostatic conditions but effectively prevented TGF-β-mediated suppression, leading to enhanced in vivo anti-tumor activity. Similarly, adoptive NKs carrying BATF deletion exhibited increased resistance to TGF-β and provided superior tumor burden control in murine AML models [28].

Beyond the TGF-β axis, targeting ZHX2 has been shown to generate more functional and persistent NKs in different mouse models [29]. Adoptive transfer of *Zhx2*^KO^ NKs suppressed hepatocellular carcinoma growth, lung melanoma metastasis, drastically improving mouse survival.

Furthermore, complementing the evidence from pharmacological inhibition, adoptive transfer of *Hif1a*^KO^ NKs demonstrated a superior ability to control lymphoma cell growth in xenograft models compared to control NKs [57]. These findings indicate a cell-intrinsic role of HIF1α in restraining NK effector functions within the TME.

To improve the efficacy of CAR-NKs and rescue TME-induced dysfunction, CRISPR-Cas9-mediated KO of CREM was shown to prevent CAR-NK exhaustion in xenograft models of both hematological and solid tumors, improving tumor control and NK persistence [64]. Similarly, IRF4 deletion effectively reversed exhaustion of adoptive NKs within metastatic melanoma models, restraining IC expression [95].

Beyond gene KO, an alternative strategy involves the activation or overexpression of a TF to potentiate the in vivo efficacy of adoptive NKs. However, the delivery of functional TF proteins remains technically demanding due to challenges of insufficient nuclear translocation, protein instability, and low target cell specificity.

Despite these issues, the forced expression of EOMES, but not of T-BET, was sufficient to enhance tumor recruitment and NK-mediated cytotoxicity, drastically reducing tumor growth in murine lymphoma models [82].

Similarly, BCL11B overexpression in induced pluripotent stem cell (iPSC)-derived NKs improved their cytotoxic profile, effectively restraining ovarian cancer growth, and prolonging median survival [53].

To our knowledge, no clinical trials are currently evaluating adoptive NKs or CAR-NKs specifically engineered with TF modulation. Given that TFs act as master regulators of multiple signaling pathways, their targeted manipulation represents a high-impact opportunity to amplify the anti-tumor response of NK-based therapies. Nevertheless, the technical difficulties in achieving efficient and stable NK genome editing currently restrain clinical feasibility. Further optimization of the engineering protocols is required to bridge the gap between preclinical success and clinical application.

## Concluding remarks

The multifaced and dynamic roles of distinct TFs govern the kinetics of NK activation, encompassing tissue recruitment, target recognition, the release of lytic granules and cytokines, as well as the transition into cancer-associated dysfunctional states. Rather than acting alone, each factor operates within a sophisticated network of transcriptional circuits. This constant interplay between activators and repressors integrates and balances extrinsic signals deriving from the TME. Consequently, time- and context-dependent transcriptional mechanisms dictate the intrinsic regulation of NK effector functions. While targeting specific TFs remains technically challenging, modulating their expression, either by pharmacological targeting or a genome editing approach, represents a promising strategy to enhance the efficacy and persistence of adoptive NK-based cell therapies in both hematological and solid tumors.

## Data Availability

No datasets were generated or analysed during the current study.

## Abbreviations

25-HC: 25-hydroxycholesterol (25HC)
ADCC: Antibody-dependent cell-mediated cytotoxicity
AHR: Aryl hydrocarbon receptor
AML: Acute myeloid leukemia
ATAC-seq: Assay for transposase-accessible chromatin sequencing
BACH2: BTB domain and CNC homolog 2
BATF: Basic leucine zipper ATF-like transcription factor
BCL11B: B cell lymphoma/leukemia 11B
BET: Bromodomain and extra-terminal domain
BETi: BET inhibitor
BLIMP1: B-lymphocyte-induced maturation protein 1
BRD4: Bromodomain-containing protein 4
CAR: Chimeric antigen receptor
CCL: C-C motif chemokine ligand
CCR7: C-C motif chemokine receptor 7
ChIP: Chromatin immunoprecipitation
ChIP-seq: Chromatin immunoprecipitation sequencing
cNK: Conventional natural killer cell
CREM: CAMP-responsive element modulator
CTLA-4: Cytotoxic T-lymphocyte associated protein 4
CXCL: C-X-C motif chemokine ligand
CXCR: C-X-C motif chemokine receptor
DAG: Diacylglycerol
DC: Dendritic cell
DGKα: Diacylglycerol kinase alpha
DNAM1: DNAX accessory molecule 1
EGR: Early growth response
ETS1: ETS proto-oncogene 1
FASL: FAS Ligand
FOXO: Forkhead box O protein
GATA-3: GATA-binding protein 3
GM-CSF: Granulocyte-macrophage colony-stimulating factor
GSK3: Glycogen synthase kinase 3
GZM: Granzyme
HIF1α: Hypoxia-inducible factor 1 subunit alpha
HOBIT: Homolog of Blimp-1 in T cells
IC: Immune checkpoint
ICER: Inducible cAMP early repressor
IFN: Interferon
iKIRs: Inhibitory killer cell immunoglobulin-like receptors
IKZF1: IKAROS family zinc finger 1
IL: Interleukin
ILC: Innate lymphoid cells
iPSC: Induced pluripotent stem cells
IRF: Interferon regulatory factor
KD: Knockdown
KIRs: Killer cell immunoglobulin-like receptors
KLF: Krüppel-like factor
KO: Knockout
LAG3: Lymphocyte-activation gene 3
MAPK: Mitogen-activated protein kinase
MEF2C: Myocyte-specific enhancer factor 2 C
MHC-I: Major histocompatibility complex class I
MM: Multiple myeloma
MMP: Matrix metalloproteinase
NET: Neutrophil extracellular trap
NFAT: Nuclear factor of activated T cells
NF-κB: Nuclear factor kappa-light-chain-enhancer of activated B cells
NK: Natural killer
NR2F6: Nuclear receptor subfamily 2 group F member 6
NSCLC: Non-small cell lung cancer
OXPHOS: Oxidative phosphorylation
PA: Phosphatidic acid
PD-1: Programmed cell death protein 1
PGE2: Prostaglandin E2
PRF1: Perforin 1
RNA-seq: RNA sequencing
RORα: Retinoid-related orphan nuclear receptorα
RUNX: Runt-related transcription factor
S1PR: Sphingosine-1-phosphate receptor
scRNA-seq: Single-cell RNA sequencing
siRNA: Short-interference RNA
SREBP1: Sterol regulatory element-binding protein 1
STAT3: Signal transducer and activator of transcription 3
T-ALL: T cell acute lymphoblastic leukemia
TF: Transcription factor
TGF-β: Transforming growth factor beta
TIGIT: T cell immunoreceptor with Ig and ITIM domains
TIM3: T cell immunoglobulin and mucin domain-containing protein 3
TIME: Tumor immune microenvironment
TiNK: Tumor-infiltrating natural killer cell
TKI: Tyrosine kinase inhibitor
TME: Tumor microenvironment
TNF: Tumor necrosis factor
TOX: Thymocyte selection-associated high mobility group box
TRAIL: Tumor-necrosis factor related apoptosis-inducing ligand
trNK: Tissue-resident natural killer cell
XBP1: X-box binding protein 1
ZHX2: Zinc fingers and homeoboxes 2
